# LIN28B Promotes Colon Cancer Migration and Recurrence

**DOI:** 10.1371/journal.pone.0109169

**Published:** 2014-10-31

**Authors:** Minghui Pang, Gang Wu, Xiaolin Hou, Nengyi Hou, Liqin Liang, Guiqing Jia, Ping Shuai, Bin Luo, Kang Wang, Guoxin Li

**Affiliations:** 1 Department of General Surgery, Sichuan Academy of Medical Sciences & Sichuan Provincial People's Hospital, Chengdu, People's Republic of China; 2 Department of Health Management Centre, Sichuan Academy of Medical Sciences & Sichuan Provincial People's Hospital, Chengdu, People's Republic of China; 3 Department of General Surgery, Nanfang Hospital, Southern Medical University, Guangzhou, People's Republic of China; Taipei Medical University, Taiwan

## Abstract

LIN28B is involved in “stemness” and tumourigenesis by negatively regulating the maturation of *let-7* microRNA family members. In this study, we showed that LIN28B expression promotes migration and recurrence of colon cancer. Immunohistochemistry and reverse-transcription polymerase chain reactions were performed to detect LIN28B expression in colon cancer tissue microarrays, paraffin-embedded surgical resected tissues and cancer cells. Loss-of-function, migration and proliferation analyses were performed to delineate the potential roles of LIN28B in colon cancer. LIN28B was upregulated in colon cancer tissue compared to normal mucosa, and its overexpression correlated with reduced patient survival and increased tumour recurrence. LIN28B suppression inhibited the migration of SW480 colon cancer cells and facilitated the cytotoxicity induced by oxaliplatin in SW480 and HCT116 colon cancer cells. In conclusion, LIN28B overexpression contributes to colon tumourigenesis, and LIN28B may serve as a diagnostic tool and therapeutic target for colon cancer.

## Introduction

*LIN28* was initially identified in *C. elegans* and was shown to be responsible for the timing of development [1]. LIN28 induces pluripotency when expressed in somatic fibroblasts along with *OCT4*, *SOX2* and *KLF4*. *LIN28B*, as a homologue of *LIN28*, was first cloned from and shown to be overexpressed in human hepatocellular carcinoma cells and clinical samples in 2006 [2]. LIN28B is a developmentally regulated RNA-binding protein that promotes tumourigenesis by blocking the post-transcriptional processing of the tumour-suppressive pri-/pre-*let-7* microRNA [3], [4]. The ability of LIN28B to regulate *let-7*, a *bona fide* tumour-suppressor, is consistent with its developmental role in regulating cell proliferation and differentiation. Interestingly, several studies revealed that *LIN28B* itself is also a target of *let-7* family members, including miR-125b [5]. Besides *let-7*, LIN28B may also bind the mRNA of intestinal stem cell markers, such as *LGR5* and *PROM1* [6]. In summary, LIN28B promotes transformation primarily by suppressing *let-7*. Competition between LIN28B and *let-7* may be critical for normal cell biology and may accelerate tumour development once this balance is perturbed.

*LIN28B* is hypermethylated in somatic tissues [7], and its aberrant reactivation may promote tumourigenesis [8]. LIN28B is frequently overexpressed in multiple cancers, especially advanced types such as progressive hepatocellular carcinoma [2], [9], epithelial ovarian cancer [10], Wilm's tumour and germ cell tumours [9]. In addition to being targeted by antecedent microRNAs, LIN28B is activated by several oncogenic pathways. C-/N-Myc induce LIN28B expression in multiple human and mouse tumour models, resulting in *let-7* repression and cell proliferation [11], [12]. *MYCN* amplification also results in LIN28B overexpression [10], and it has been reported that c-Myc is related to sporadic large bowel cancer and familial polyposis coli [13], implying a role for LIN28B in colon cancer.

Despite great achievements in surgery, chemotherapy and the development of novel molecular-targeted drugs, such as bevacizumab (Avastin), the incidence of colon cancer continues to increase [14]. Each year, tumours of the colon are responsible for 655, 000 deaths globally. Because *Let-7* functions as a potential growth suppressor in human colon cancer cells [15], we examined LIN28B expression in colon cancer tissues to determine the balance between LIN28B and *let-7* expression. We examined LIN28B expression in human colon cancer tumours via tissue microarray and found that LIN28B was significantly upregulated in tumour tissue compared to normal colonic mucosa. To further analyse the survival of patients with colon cancer, we selected an additional cohort of postoperative patients with detailed pathology records and follow-up data from 2004 to 2009. As anticipated, LIN28B overexpression correlated with reduced patient survival and an increased likelihood of tumour recurrence. Furthermore, we found that silencing LIN28B inhibited the migration of SW480 cells and sensitised SW480 and HCT116 colon cancer cells to oxaliplatin-induced cytotoxicity.

## Patients and Methods

### Tumour tissue analysis

Samples of human colon carcinomas and normal colons were obtained from a human colon carcinoma tissue array (CO2161; US Biomax), which contained 204 adenocarcinomas, 4 signet ring cell cancers and 8 normal colon mucosa samples. Detailed clinical information, including differentiation status and TNM grading, were also provided for each sample. An additional cohort of 149 colon carcinoma samples was collected from specimens that had been surgically resected in 2004 from consenting patients at Nanfang Hospital (Southern Medical University, Guangzhou, China), according to an Institutional Review Board–approved protocol. Each of these cases was followed, in detail, through June 2009. We confirmed the TNM grade for samples from both cohorts. In total, 357 colon cancer tumours and 8 normal colon samples were used for the IHC analysis. The detailed information for all tumours and patients is provided in Table 1. The study protocol was approved by the ethics review board of Nanfang Hospital. We have obtained written informed consent from all study participants. All of the procedures were done in accordance with the Declaration of Helsinki and relevant policies in China.

**Table 1:** Clinical characteristics of patients/tumours at the time of diagnosis.

Clinical variants	Tissue microarray (n = 208)	Surgically resected tumours (n = 149)
Average age (years)	54.80	56.9
Sex		
male	118	88
female	90	61
Pathology grade	17 cases not classified	
moderately/well-differentiated	150	137
poorly differentiated	41	12
TNM grade		
I	22	26 (1 Tis)
II	128	65
III	47	42
IV	11	16
Tumour type		
adenocarcinomas	204	148
signet ring cell cancers	4	1

Two pathologists scored the LIN28B staining intensity according to the following scale: a score of 0/1 was used to signify weak/low LIN28B intensity (0–25% positive rate); a score of 2 represented intermediate intensity (25–50% positive rate); and a score of 3 was used for high-intensity staining (>50% positive rate). Tumours of grades 1, 2 and 3 are equivalent to tumours classified as well differentiated, moderately differentiated or poorly differentiated, respectively, using microscopy.

Log rank tests (Mantel–Cox and Breslow) were performed to compare the survival and recurrence distributions of the different intensity groups (0–2 vs. 3). The correlation between the staining intensity and survival or recurrence was determined using a chi-square analysis, and a 95% confidence interval was calculated to determine statistical significance.

### Cell culture

The SW480, Caco2 and HCT116 colon cancer cell lines were purchased from ATCC (American Type Culture Collection, Manassas, VA) and were cultured in Dulbecco's modification of Eagle's medium (DMEM; Gibco, Carlsbad, CA, USA) supplemented with 10% foetal bovine serum (FBS; HyClone, South Logan, UT, USA). All cell lines were maintained in a 5% CO_2_, humidified atmosphere.

### Oligoribonucleotides

A LIN28B-specific siRNA and a negative control small RNA (NC) were constructed by Genepharma (Shanghai, China). LIN28B- and GAPDH-specific primers were synthesised by TaKaRa (TaKaRa Biotechnology, Dalian, China).

### Cell transfection

RNA oligoribonucleotides were transfected using Lipofectamine 2000 (Invitrogen), according to the manufacturer's instructions. Between 50 and 100 nM of the RNA oligoribonucleotides were used for each transfection.

### Semi-quantitative reverse-transcription polymerase chain reaction (RT-PCR) and quantitative reverse-transcription polymerase chain reaction (qRT-PCR)

Total RNA was extracted using TRIzol reagent (Invitrogen) and was then treated with DNase I (Tiangen Biotech, Beijing, China). The cDNA for the qRT-PCR was synthesised with the PrimeScript RT reagent kit and the gDNA Eraser (TaKaRa). The PCR amplification procedure for GAPDH was as follows: 94°C for 3 min; 30 cycles of 94°C for 30 s, 60°C for 30 s and 72°C for 30 s; and a terminal elongation at 72°C for 5 min. The amplification of LIN28B was performed as follows: 95°C for 2 min; 30 cycles of 95°C for 30 s, 58°C for 30 s and 72°C for 30 s; and a final step at 72°C for 5 min. The qRT-PCR assays were performed using the SYBR PrimeScript RT-PCR kit (TaKaRa) for GAPDH and LIN28B.

### Immunoblots

Cytosolic protein fractions were prepared using RIPA buffer (Beyotime Biotechnology, Haimen, China). The antibodies used for the immunoblots were specific for LIN28B (1∶10, ab71415; Abcam, Cambridge, MA, USA) and GAPDH (G9295; Sigma-Aldrich, USA). Western blots and IHC were performed according to standard procedures.

### Migration analysis

SW480 cells (1×10^4^ cells in 100 µl serum-free medium), which had been transfected with the NC or si-LIN28B, were placed in the top chamber of Transwell culture dishes (8 µm; BD Biosciences, San Jose, CA). The lower chamber was filled with 600 µL of conditioned medium. After 24 hours, the cells that had not migrated to the lower chamber were removed from the upper surface of the Transwell membrane with a cotton swab. Migrated cells on the lower membrane surface were fixed, stained with 0.1% crystal violet and imaged.

### Cell viability analysis

SW480 or HCT116 cells, which had been transfected with either the NC or si-LIN28B, were seeded in triplicate in 96-well plates at a concentration of 1×10^4^ cells per well. After the cells had adhered to the plates, they were incubated with different concentrations of oxaliplatin (e.g., 100, 10, 1, 0.1, 0.01 and 0.001 µg/mL; Jiangsu Hengrui Medicine Co., Ltd., Jiangsu, China) for 4 hours and were then transferred to complete medium for an additional 20 hours. At the indicated time point, 10 µL of CCK-8 solution (Dojindo, Kumamoto, Japan) was added to each well, and the cells were incubated for 3 h at 37°C. The absorbance (A) was recorded at 450 nm using a plate reader. The experiment was performed in triplicate, and the cell inhibition ratio was determined using the following equation: (1 - test group A/control group A) ×100%. The IC_50_ (50% inhibiting concentration) value was calculated using specific software (LOGIT method).

### Statistical analysis

A log rank test was performed to compare the survival and recurrence distributions for the various intensity groups (0–2 vs. 3). The correlation between staining intensity and survival or recurrence was determined according to a chi-square analysis, where p values less than 0.05 were considered to be statistically significant. A 95% confidence interval was calculated for confirmation of statistical significance.

## Results

### LIN28B was upregulated in colon carcinomas

**Figure 1:**
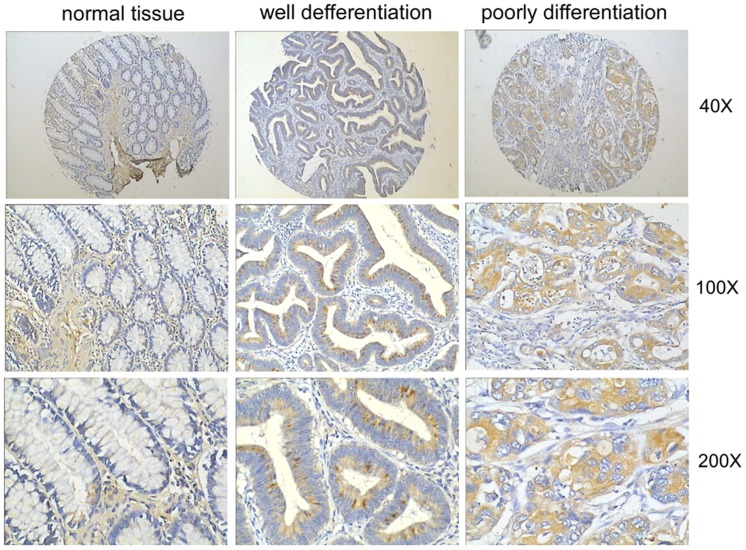
LIN28B is significantly overexpressed in colon tumour tissues. IHC showed that LIN28B was markedly upregulated in tumour tissues compared with the normal mucosa, which demonstrated very little LIN28B expression. Representative graphs are presented.

To examine the potential roles for LIN28B in colon cancer, we first compared LIN28B protein expression between 208 tumour samples and 8 non-matched normal mucosa samples from a tissue microarray using IHC. The staining intensity of LIN28B was scored by two pathologists who were blinded to the clinical information. Consistent with previous reports [6], [16], LIN28B was significantly overexpressed in tumour tissues compared to normal tissues (Fig. 1, p<0.001).

### LIN28B overexpression correlated with reduced patient survival and a high risk of recurrence

**Figure 2:**
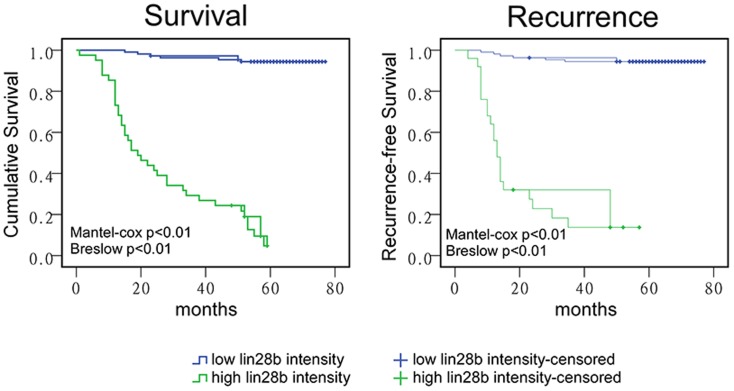
LIN28B overexpression correlated with reduced patient survival and an increased likelihood of tumour recurrence. (A) Higher LIN28B staining intensity from stage I, II and III colon cancers correlated with reduced patient survival. (B) High LIN28B expression was related to a higher probability of tumour recurrence (p<0.01; log rank test).

Data from patients who underwent surgery (TNM I-III, paraffin-embedded surgical resected tissues) were further analysed via log rank tests to investigate the influence of LIN28B overexpression on patient survival and recurrence. This analysis revealed a correlation between low-intensity LIN28B staining from samples of TNM grade I and II tumours and increased patient survival (Mantel-Cox p<0.01; Breslow, p<0.01) and a lower likelihood of tumour recurrence (Mantel-Cox p<0.01; Breslow p<0.01) (Fig. 2).

Taken together, our results reveal that LIN28B expression is closely related to overall patient survival and recurrence of colon cancer.

### LIN28B loss-of-function sensitised SW480 and HCT116 cells to oxaliplatin-mediated cytotoxicity

**Figure 3:**
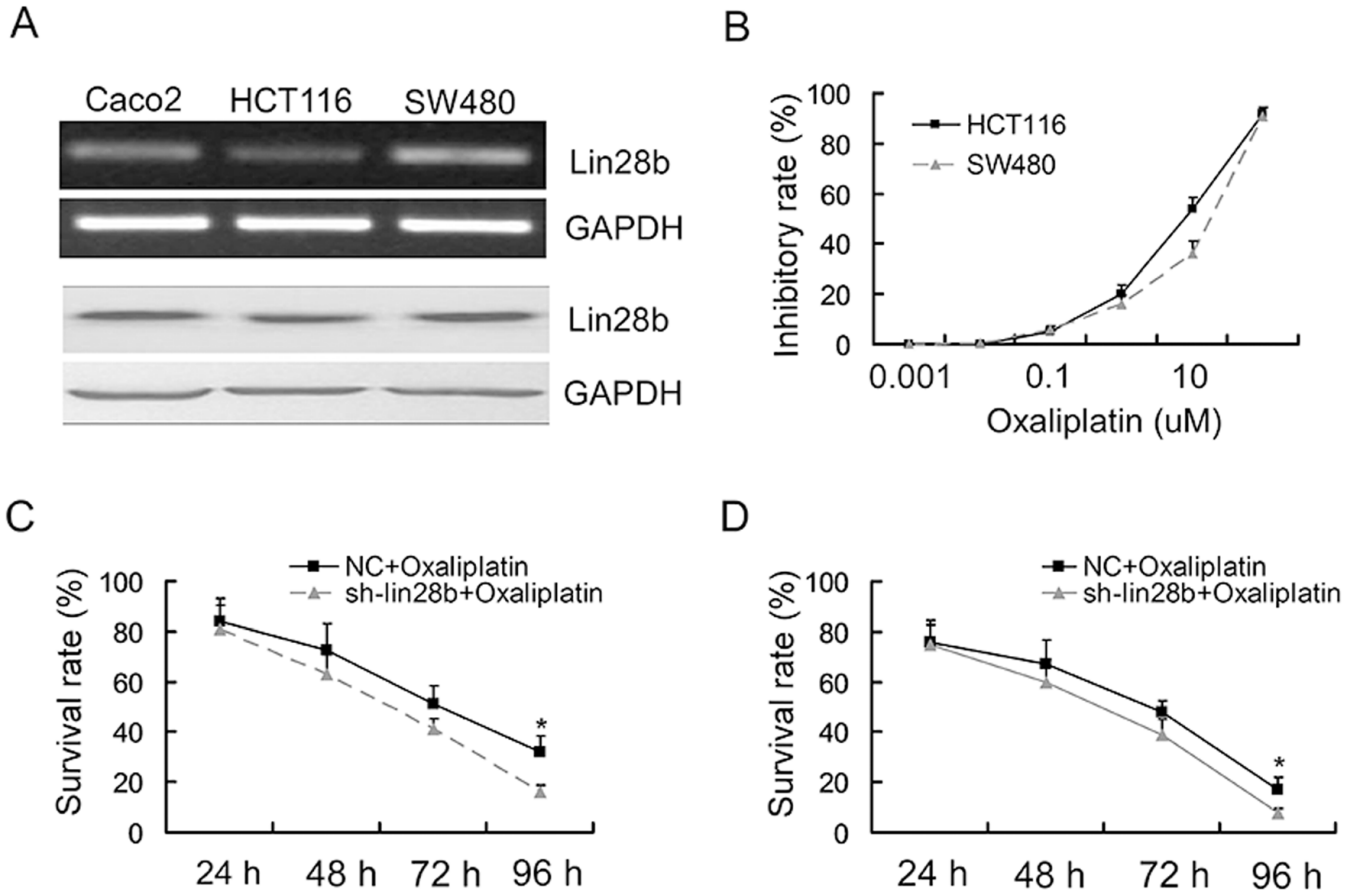
Repression of LIN28B sensitised SW480 and HCT116 cell to oxaliplatin-induced cytotoxicity. (A) RT-PCR and Western blot analyses evaluated LIN28B expression levels in Caco2, SW480 and HCT116 cells. (B) SW480 and HCT116 cells were plated in 96-well plates and were incubated with the indicated concentrations of oxaliplatin. Cell viability was assessed using a CCK-8 assay kit after 72 h of exposure to the drug or diluent control. IC_50_ values were calculated after curve fitting using the XLfit software. (C–D) HCT116 and SW480 cells were transfected with NC or si-LIN28B 24 h prior to oxaliplatin (IC_50_ value) treatment. The inhibitory rate, normalised to NC, was calculated using CCK-8 absorbance at the indicated time points. The data are represented as the mean fold change ± SE (n = 3; Student's t-test).

**Figure 4:**
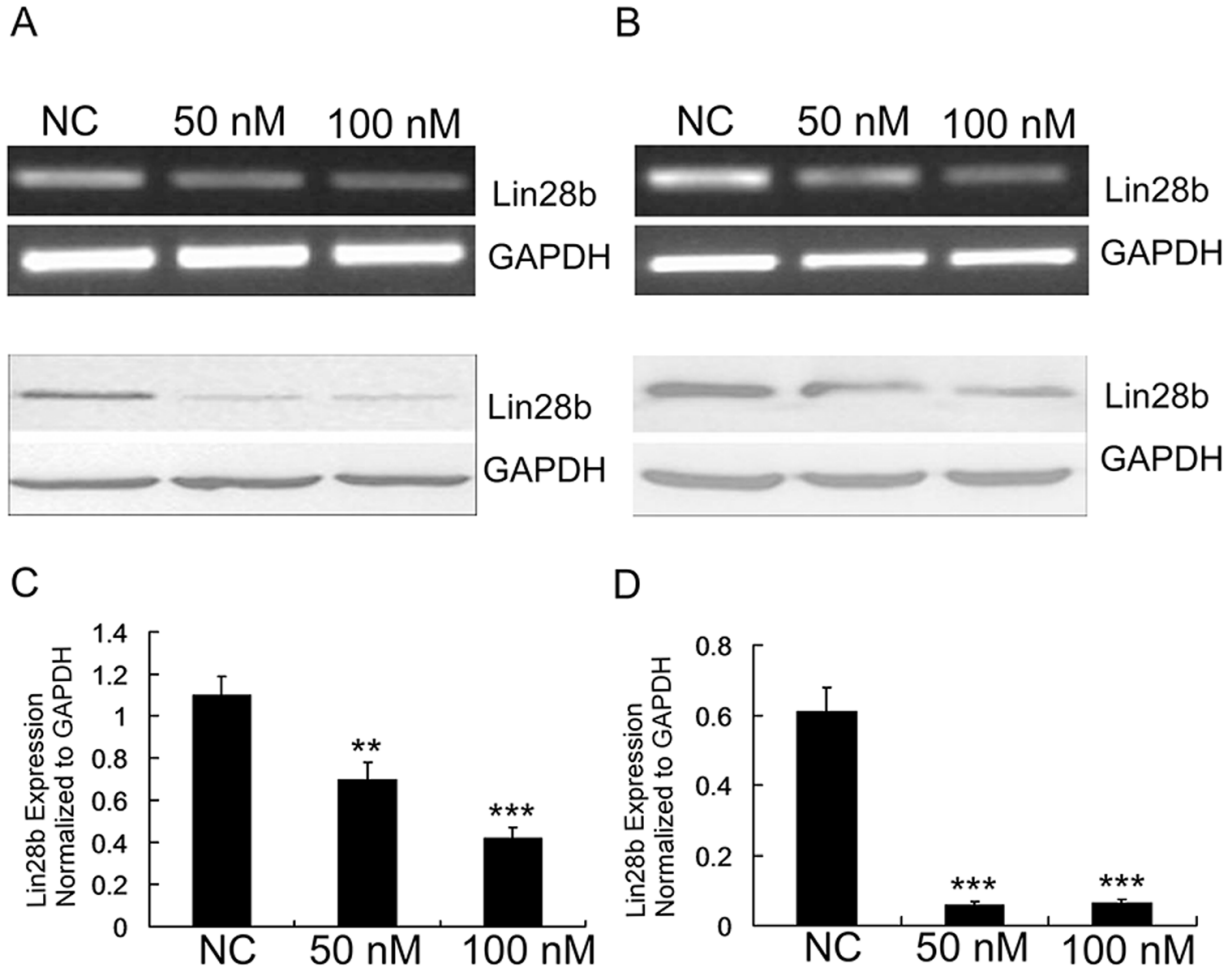
si-LIN28B efficiently inhibited the expression of LIN28B in HCT116 and SW480 cells. (A–B) RT-PCR and Western blot analyses confirmed the efficiency of sh-LIN28B knockdown in HCT116 and SW480 cells. (C–D) A qRT-PCR analysis also confirmed the downregulation of LIN28B mRNA in these cells. The data are represented as the mean ± SE (n = 3; Student's *t*-test).

The *let-7* tumour suppressor microRNA family members are known to regulate chemosensitivity [17], while LIN28B promotes malignancy mainly by inhibiting *let-7* biogenesis. Thus, we sought to determine whether LIN28B could influence chemosensitivity to oxaliplatin. We examined LIN28B expression in three colon cancer cell lines using RT-PCR and Western blotting (Fig. 3A). HCT116 and SW480 cells were selected for use in these experiments due to their low and high level of Lin28 expression, respectively. Oxaliplatin induced concentration-dependent cytotoxicity in these cells (Fig. 3B). The IC_50_ value for oxaliplatin was 6.23±0.75 µg/mL for the HCT116 cells, and this was increased by 30% to 10.7±2.26 µg/mL in the SW480 cells. This finding suggested that differences in LIN28B expression may serve to modulate chemosensitivity in colon cancer cells. To verify this hypothesis, we constructed LIN28B-specific siRNA and control small RNA. The efficiency of the si-LIN28B was determined in both cell lines (Fig. 4A–D), and then a CCK-8 analysis was performed to explore the interactions between the si-LIN28B and oxaliplatin in HCT116 and SW480 cells. The results indicated a synergistic effect between si-LIN28B and oxaliplatin (Fig. 3C–D), which suggests that the targeting of LIN28B may be capable of sensitising colon cancer cells to oxaliplatin therapy.

### Downregulation of LIN28B repressed the migration of SW480 cells

**Figure 5:**
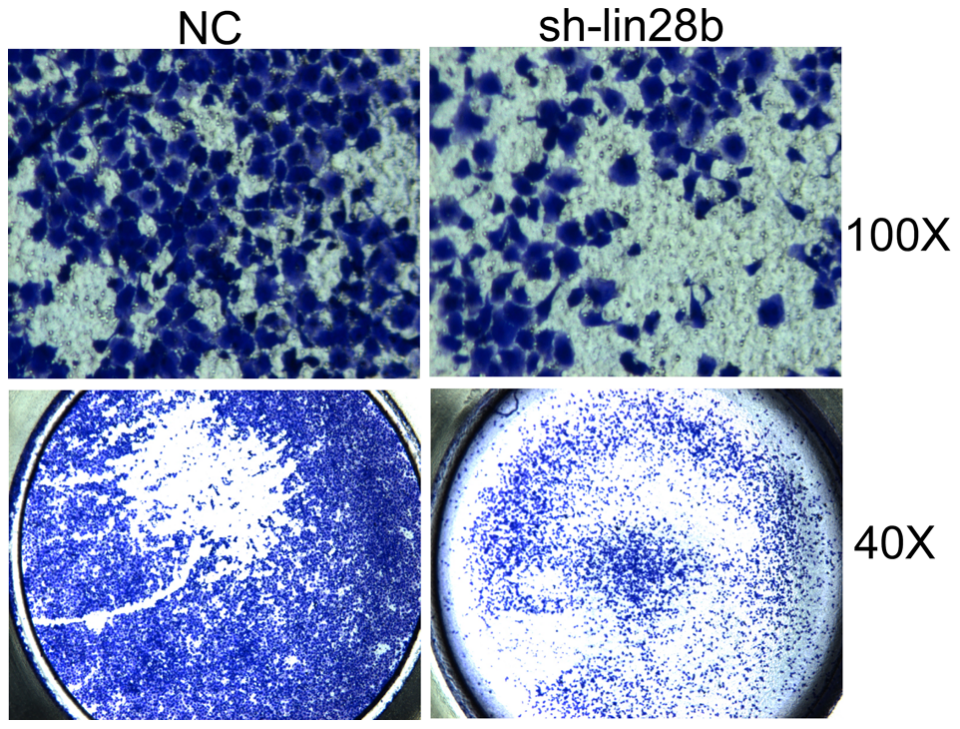
Silencing of LIN28B suppressed the migration of SW480 cells. The cells were transfected with 50 nM NC or si-LIN28B and were allowed to migrate through a Transwell chamber. Representative graphs are presented.

As LIN28B overexpression was correlated with tumour recurrence and patient survival, we next sought to explore whether LIN28B expression influences the migration of colon cancer cells. We knocked down LIN28B expression using siRNA, and the Transwell analysis indicated that suppression of LIN28B significantly inhibited the migration of SW480 cells (Fig. 5).

## Discussion

Several stemness-related genes, which are referred to as oncofoetal genes, are thought to promote tumourigenesis upon re-expression in somatic cells. The presence of CD133^+^ cancer stem cells or cancer-initiating cells may explain the metastasis, recurrence and chemo-resistance of colon cancer [18], [19]. *Let-7* is involved in regulating the self renewal and tumourigenicity of breast cancer-initiating cells [20] and is also repressed in colon cancer cells [15]. LIN28B overexpression is known to contribute to carcinogenesis by blocking the biogenesis of *let-7*, which prompted us to evaluate whether the balance between tumour-promoting LIN28B and tumour-suppressive *let-7* was altered in colon cancer [21]. We found that LIN28B expression was upregulated in colon tumour tissues, and this expression correlated with reduced patient survival, and a higher probability of recurrence. Additionally, the silencing of LIN28B sensitised colon cancer cells to cytotoxicity induced by oxaliplatin and inhibited their migration *in vitro*.

*Lin28* is expressed in various stem cell populations, although its expression in terminally differentiated somatic tissues is scarce. Recently, when expressed in combination with *OCT4*, *NANOG* and *SOX2, Lin28* was shown to act as a replacement for *c-Myc* to reprogram human somatic fibroblasts to pluripotency [22]. As a homologue of Lin28, *LIN28B* has primarily been cloned from hepatocellular carcinomas, and its overexpression has been shown to promote cancer cell proliferation [2]. The activation of LIN28B results in reduced repression of *let-7* targets, such as *HMGA2*, *Dicer1* [10], *KRAS* [23] and *c-Myc*, and the activation of corresponding oncogenic signalling pathways. Interestingly, *LIN28B* and its upstream regulator *c-Myc* [12] are also targets of *let-7*; consequently, a *c-Myc-LIN28B-let-7* axis, or feedback loop, which may serve to repress tumour growth, may exist. Thus, the loss of control over this axis may accelerate tumour growth.

As mentioned previously, *LIN28B* is regulated by several oncogenic factors; members of the *MYC* family, including *C-Myc*, *N-Myc* and *MYCN*, can activate LIN28B, which suggests that *LIN28B* is an important target of *MYC* and may explain why *LIN28B* can replace *c-Myc* for the reprogramming of somatic cells into pluripotency. The *c-Myc* transcription factor can be activated by the *Wnt/APC* pathway, which is deregulated in colon cancer [24]. *APC* gene alterations, if not inherited, represent the earliest molecular alterations during the development of colorectal cancer, whereas structural alterations of the *myc, ras, p53*, *MCC* and *DCC* genes are considered to represent late events [25]. In summary, our findings reveal the existence of a preliminary *Wnt/APC*-*Myc*-*LIN28B*-*let-7* pathway in colon carcinogenesis. We hypothesise that *Wnt/APC*-*Myc* pathway activation is the initiating event and that the *LIN28B-let-7-c-Myc/LIN28B* feedback loop accelerates colon carcinogenesis. However, further studies are required to verify the role of this feedback loop in carcinogenesis.

Metastasis and recurrence are the primary reasons for the decrease in overall survival among patients with malignant tumours. Thus, it is of great interest to be able to predict tumour metastasis and recurrence at an early stage. Furthermore, predicting tumour recurrence for stage II/III patients may guide adjuvant chemotherapy. Improved methods for identifying stage II patients at a high risk of recurrence [26], which are based on the unique characteristics of each individual tumour, may result in thousands of lives saved each year. In addition, techniques for identifying stage III patients who are at a low risk for disease recurrence after surgery may spare these individuals from the cost, time and toxicity associated with chemotherapy [27]. In our study, LIN28B staining intensity (>50%) correlated with reduced patient survival. Importantly, LIN28B expression could predict the recurrence of TNM grade II/III tumours after surgical resection.

While this manuscript was in preparation, a study from King et al. reported that LIN28B promotes colon cancer progression and metastasis both *in vivo* [16] and *in vitro* through *let-7*-dependent and -independent mechanisms [6]. Our data are consistent with these findings, and we also suggest that detecting the expression of LIN28B might aid in determining the appropriate scheme of adjuvant chemotherapy in patients with TNM grade II and III colon carcinomas.

In conclusion, our data suggest an important role for *LIN28B* in colon carcinogenesis. LIN28B expression was shown to predict patient survival, which implies that *LIN28B* may be a useful target for novel molecular therapeutics.
